# Impact of accelerated weather aging on building energy efficiency using cement and gypsum boards with shape-stabilized phase change materials

**DOI:** 10.1038/s41598-025-24728-8

**Published:** 2025-11-19

**Authors:** Filippos Lygerakis, Christina Gioti, Michael A. Karakassides, Dimitriοs P. Gournis, Ioannis V. Yentekakis, Dionysia Kolokotsa

**Affiliations:** 1 School of Chemical and Environmental Engineering, Technical University of Crete, 73100 Chania, Greece; 2 Department of Materials Science & Engineering, University of Ioannina, 45110 Ioannina, Greece; 3 Institute of Geoenergy/Foundation for Research & Technology-Hellas (IG/FORTH), 73100 Chania, Crete, Greece

**Keywords:** PCMs, Accelerated weather aging, Building energy savings, Cement samples, Gypsum samples, Octadecane, Energy science and technology, Engineering, Materials science

## Abstract

This study evaluates the performance and durability of phase-change material (PCM)-enhanced gypsum and cement boards subjected to accelerated weather aging, and their subsequent impact on building energy efficiency. Gypsum and cement boards containing 10–30% shape-stabilized PCM (ss-PCM) were exposed to controlled environmental stress. Thermal conductivity, specific heat, emissivity, solar reflectance, and latent heat storage capacity were measured before and after aging. Prior to aging, gypsum boards achieved up to 5.4% net annual energy savings, and cement boards up to 4.8%. Post-aging, gypsum samples exhibited a near-complete loss of latent heat capacity, reducing energy savings to 0.7%. In contrast, cement boards with 30% ss-PCM retained approximately 54% of their original latent heat, retaining 3.1% net energy savings despite a 35% reduction relative to pre-aging performance. Solar reflectance increased in gypsum boards (up to 38.8%) after aging, while thermal conductivity remained stable or improved in cement boards. Emissivity values remained high (> 0.90) across all samples, with only minor deviations after aging. These findings highlight the relative environmental durability of PCM-enhanced cement boards and highlighting the influence of matrix composition and PCM encapsulation on sustaining energy performance under accelerated aging conditions.

**Supplementary Information:**

The online version contains supplementary material available at 10.1038/s41598-025-24728-8.

## Introduction

PCMs have emerged as a promising passive solution for enhancing building energy efficiency and indoor thermal comfort. By absorbing and releasing latent heat during phase transitions, PCMs increase the thermal inertia of building envelopes, effectively reducing indoor temperature fluctuations and peak heating or cooling demands. Their integration into walls, ceilings, or construction elements is especially beneficial in regions with large diurnal temperature swings, such as the Mediterranean, where paraffin-based PCMs like n-octadecane, with a melting point of 25–30 °C, align well with indoor comfort temperatures^1–4^. Studies have reported that PCM-enhanced gypsum and cementitious materials can significantly lower cooling loads and stabilize indoor conditions in Mediterranean climates^3,4^.

Despite these advantages, the long-term performance and durability of PCM-integrated materials remain under-explored. PCMs in real-world applications experience repeated thermal cycling, UV exposure, and humidity variations, all of which may induce chemical and physical degradation. Reported concerns include loss of latent heat capacity, leakage, supercooling, and damage to encapsulation structures^5^. While form-stable composites and encapsulated systems offer improved resilience, there is growing but still incomplete understanding of how these materials perform under prolonged environmental exposure. Ensuring thermal effectiveness over time is critical for reliable PCM deployment in building applications.

Several studies have sought to enhance PCM stability using porous matrices or lightweight aggregates that prevent leakage and stabilize the thermal behavior of PCMs^6,7^. In addition to their thermal energy storage capacity, PCM composites can provide multifunctionality when stabilized within carbonaceous matrices. Specifically, the incorporation of activated carbon, carbon foams, or expanded graphite not only enhances the structural stability of the PCM but also imparts electromagnetic shielding properties, a synergistic effect that has been recently emphasized in the literature^8–10^. Regarding stability, Wadee et al^5^ reported that paraffin-impregnated boards preserved their thermal conductivity and heat capacity after 700 thermal cycles, despite the pure PCM exhibiting up to 23% reduction in latent heat. Yet, such findings often rely on isolated thermal cycling or material testing, without translating degradation effects into energy performance at the building level.

Critically, few studies have systematically linked material-level durability assessments with whole-building energy modeling. While some works have simulated the energy performance of PCM-equipped buildings, they often rely on ideal or unaged material properties^11,12^. Others conduct aging tests without integrating those findings into simulation frameworks. This disconnect limits our understanding of how PCM degradation affects real-world energy savings over time. Moreover, most available studies focus on temperate or generic climate profiles, leaving Mediterranean applications underrepresented in durability-focused PCM research^13,14^.

To address this gap, the present study investigates the effects of accelerated weather aging on the performance of gypsum and cement boards enhanced with n-octadecane PCM. Using a xenon arc test chamber, several years of outdoor exposure are simulated, including UV radiation and humidity, for PCM-enhanced cement board samples and only UV radiation for PCM-enhanced gypsum board samples. The pre- and post-aging thermal and optical properties (thermal conductivity, emissivity, solar reflectance, and latent heat) are measured and then incorporated into EnergyPlus simulations of a Mediterranean climate (Csa) residential case study. An acceleration factor is used to relate chamber exposure time to real climate aging. This approach enables a direct evaluation of how weather-induced degradation in PCM properties influences the energy-saving potential of these materials in actual building scenarios.

To the authors’ knowledge, this is among the first studies to integrate standardized accelerated weather aging protocols with calibrated building energy simulations to assess the pre- and post- weather aging energy performance of PCM-integrated building envelopes. By linking material-level degradation to modeled energy impacts, this study contributes to a more robust understanding of PCM durability and supports resilient design strategies for warm-climate applications.

Table 1 summarizes recent studies on PCM-integrated building materials and highlights their scope and limitations. Several works focus on durability through artificial aging protocols, such as QUV or UV exposure^13,15,16^, but stop short of translating the degraded material properties into building energy simulations. Others^17–19^ explore EnergyPlus-based performance modeling but assume unaged PCM properties and omit durability assessment. Paolini et al.^20^ provide a valuable methodological precedent by coupling natural aging measurements of wall reflectance with EnergyPlus simulations, demonstrating the importance of integrating real-world degradation data into building modeling. More recent works have started to combine laboratory durability assessment with simulation: Liu et al.^21^ measured thermal stability and leakage resistance of PCM slurries before simulating them in EnergyPlus, while Hidalgo-Araujo et al.^22^ performed accelerated aging tests on thermochromic-PCM roof coatings and evaluated their impact on annual building energy savings. However, neither study includes a full set of thermophysical property measurements (e.g., latent heat, thermal conductivity, emissivity) nor conducts a direct pre/post-aging simulation comparison. In contrast, the present work applies xenon arc accelerated weathering following ISO 4892–2, which simultaneously exposes samples to UV light and water spray cycles, thereby reproducing key degradation mechanisms in a compressed timeframe. This approach is more severe than natural weathering and provides a worst-case benchmark for long-term performance. Furthermore, unlike most literature where post-aging measurements are rarely used in building simulation, our study feeds pre- and post-weathering data (optical, thermal, and latent heat properties) into EnergyPlus to quantify the direct impact of environmental aging on building energy performance.

**Table 1:** Comparison of key PCM studies with the present work (EP = EnergyPlus, ✓ = Included, ✗ = Not included).

Study	Pcm type	Material studied	Weather aging	energy Modeling	Climate	Key limitation
^5^	Paraffin (RT18HC/22HC/25HC)	Gypsum & cement mortars (with PCM-loaded granules)	✓ (Thermal cycling, 700 cycles)	✗	–	Only thermal cycling tested; building-level performance not evaluated
^13^	Paraffin (bulk)	PU membrane (cool roof)	✓ (QUV per ASTM G154)	✗	Csa	No energy modeling or wall materials
^15^	Paraffin-HDPE-EG	Heat absorber panels (HDPE)	✓ (UV)	✗	BWh	No simulation; different matrix (plastic); no reflectance
^16^	Microencapsulated paraffin wax PCMs	Cementitious façade plaster (exterior finish)	✓ (QUV, UV + condensation)	✗	Dfd	No simulation
^17^	Microencapsulated	Gypsum panels (internal)	✗	✓ (EP)	Cfb	No aging or optical property tracking
^18^	Bio-paraffin	PCM-integrated wall	✗	✓(EP)	Csa	No aging or durability data
^19^	n-Octadecane	Gypsum & cement boards	✗	✓(EP)	Csa	No aging or durability testing
^20^	None (cool-wall coating only)	Exterior wall coatings (no PCM)	✓ (Natural aging, multi-year exposure)	✓ (EP)	Cfa	Strong methodological precedent (pre- and post-aging data used in simulation) but not PCM-based; only optical property degradation considered
^21^	Microencapsulated PCM slurry	Gypsum board	✓(Thermal stability test + leakage resistance)	✓ (EP)	-	No weathering beyond thermal; no UV or water moisture exposure
^22^	Thermochromic + PCM (microencapsulated paraffin)	Roof coating	✓ (Accelerated aging ASTM D7897: UV + humidity + heat)	✓ (EP)	Cfa	No explicit pre/post coupling in the simulation inputs
**Current Work**	n-Octadecane	Gypsum & cement boards	✓ (xenon arc test chamber)	✓(EP)	Csa	(Fills experimental & modeling gap)

## Materials and methods

### Methodology

**Fig. 1:**
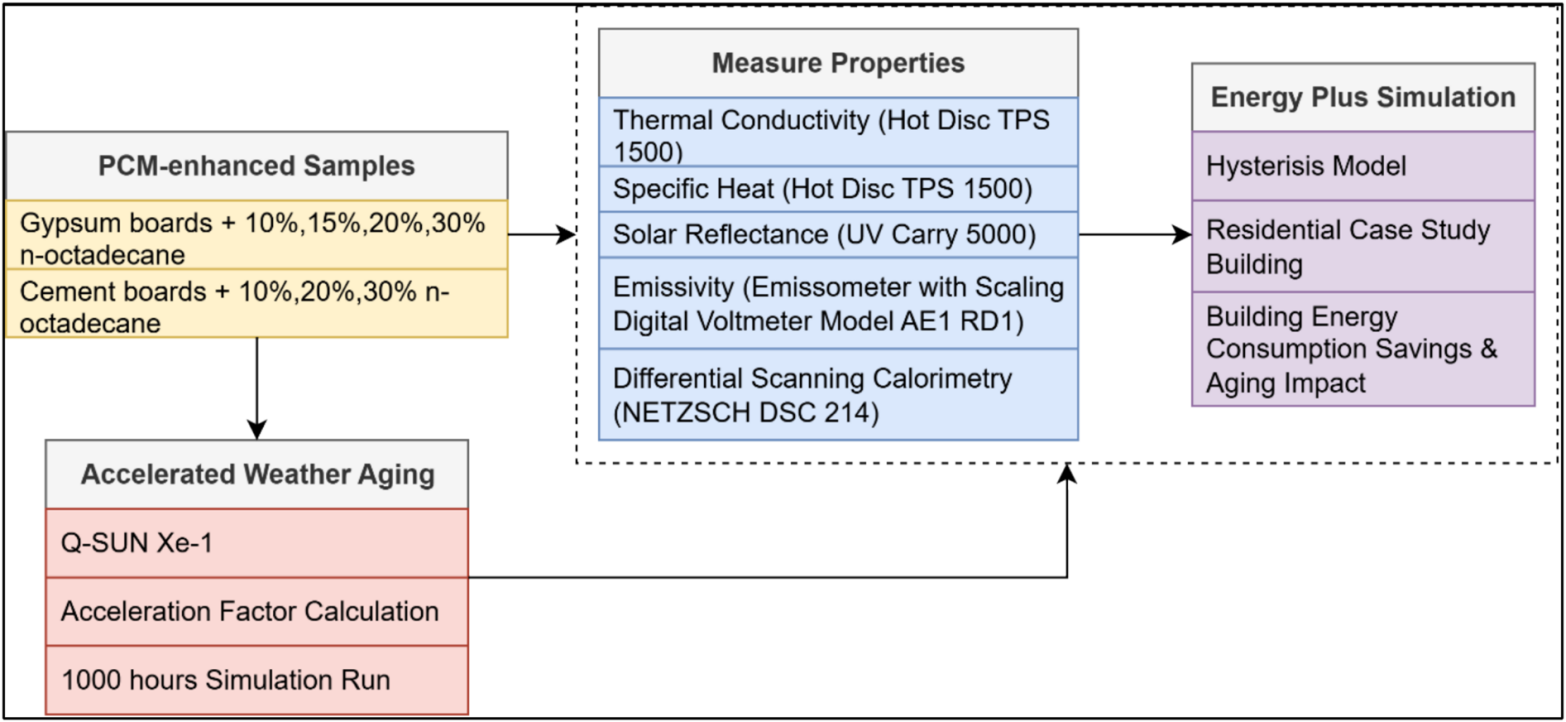
Methodology.

In Fig. 1, the methodology followed for the current work is depicted and aims to evaluate the performance and durability of ss-PCM-enhanced building materials.

The shape-stabilized PCM (ss-PCM) used in this study was prepared following the procedure described by Gioti et al.^9^. Briefly, carbon–red mud foams (CRMFoams) were synthesized by dispersing red mud in acetone, mixing with a novolac resin-hexamethylenetetramine solution, and curing the foamed mixture before carbonization at 1100 °C under N₂ to obtain a stable porous structure. The foams were then impregnated with melted n-octadecane at 90 °C under vacuum until complete pore filling was achieved, followed by cooling to lock the PCM within the matrix. Excess PCM was removed by draining, and the composites were weighed to confirm PCM loading. Afterwards, gypsum and cement board samples were prepared by incorporating varying concentrations of stabilized PCM composite. In particular gypsum boards were fabricated incorporating ss-PCM at nominal loadings of 10%, 15%, 20%, and 30% w/w, while cement boards were produced with ss-PCM contents of 10%, 20%, and 30% v/v, respectively. The actual concentrations of shape-stabilized PCM, as determined by thermogravimetric analysis (TGA), were in close agreement with the nominal compositions, although the technique may introduce some uncertainty due to the small sample quantities employed.

The properties of these samples were first measured and simulated to establish baseline performance metrics. Solar reflectance was measured once per scenario following ASTM E903-12 using a Cary 5000 UV–Vis–NIR spectrophotometer with an integrating sphere (DRA-2500). Measurements were not replicated across multiple boards; instead, instrument repeatability (± 0.01 absolute reflectance) and calibration against a Spectralon reference standard ensure reliability of the reported values. Emissivity was determined via a transient method in accordance with ASTM C1371, using a Scaling Digital Voltmeter Model AE1 RD1. Thermal conductivity and specific heat were measured using a Hot Disk TPS 1500 following ISO 22,007–2, with 20 repeated measurements per board, and reported as mean ± standard deviation. Differential Scanning Calorimetry (DSC) was conducted using a NETZSCH DSC 214 Polyma system with Concavus® aluminum crucibles (pierced lids) under a nitrogen purge (60 ml/min). Approximately 18–20 mg of material was weighed and sealed per test. The temperature program consisted of heating from 0 °C to 40 °C at 2 K/min, followed by cooling back to 0 °C at the same rate, enabling the capture of melting and solidification events. Each sample was measured in triplicate, and enthalpies of melting and freezing were obtained by numerical integration of the heat flow peaks. Peak onset, maximum, and end temperatures were extracted to determine phase change intervals. EnergyPlus simulations were then carried out using a hysteresis model to represent PCM phase-change behavior within a residential building model, quantifying baseline energy performance and consumption savings. All instruments were calibrated according to manufacturer specifications prior to testing, and measurement repeatability was within ± 3% for thermal properties and ± 0.01 absolute reflectance for SR. Subsequently, the samples underwent accelerated weather aging using a Q-SUN Xe-1 weathering chamber. In addition, an acceleration aging factor was calculated to correlate laboratory aging to real-world environmental exposure. Samples were exposed for a simulated period of 1000 h to evaluate potential PCM degradation and material durability. After the accelerated weather aging, the same critical thermal and optical properties were re-measured using identical instruments. These post-aging measurements were again integrated into EnergyPlus simulations to assess the influence of weather-induced degradation on the PCM-enhanced materials’ performance.

Samples were aged in a Q-SUN Xe-1 chamber following ISO 4892–2, using daylight filters (340 nm). The chamber-controlled irradiance and black panel temperature, while relative humidity followed ambient laboratory conditions (typically 40–60% RH). The cycle consisted of 1 h 42 min light exposure at 0.51 W/m^2^/nm (65 °C black-panel temperature), followed by 18 min of light plus water spray (5 s on, 55 s off). This sequence was repeated continuously for 1000 h. Cement boards were subjected to the full three-step cycle, whereas gypsum boards followed a light-only cycle, as initial trials with water spray led to sample dissolution and prevented post-aging measurements. ISO 4892–2 specifies spectral power distribution, irradiance level, temperature control, and wetting cycles, enabling repeatable laboratory reproduction of photolytic and moisture-related degradation. However, the protocol does not account for additional natural weathering factors such as fluctuating humidity, atmospheric pollutants, biological growth, or freeze–thaw cycles. Lamp irradiance and black-panel temperature were verified weekly per ISO 4892–2 recommendations to ensure consistent exposure conditions throughout the 1000 h test.

Finally, comparisons between pre-aging and post-aging measurements and simulation results were conducted to evaluate the impacts of weather aging on PCM effectiveness, material durability, and overall energy efficiency and thermal performance of building envelopes.

Although PCM-enhanced gypsum and cement boards were modeled as interior envelope layers shielded from direct environmental exposure, the accelerated aging protocol (ISO 4892–2) was intentionally designed as a worst-case scenario. It involves continuous UV exposure, elevated temperatures, and water spary without dark periods, simulating extreme degradation to conservatively assess long-term material performance.

Pre- and post-aging measured values for thermal conductivity, specific heat, emissivity, solar reflectance, and DSC-derived enthalpies were directly used as inputs to EnergyPlus material definitions. This ensured that the simulations capture the measured degradation effects without assumptions.

### Case study building

**Fig. 2:**
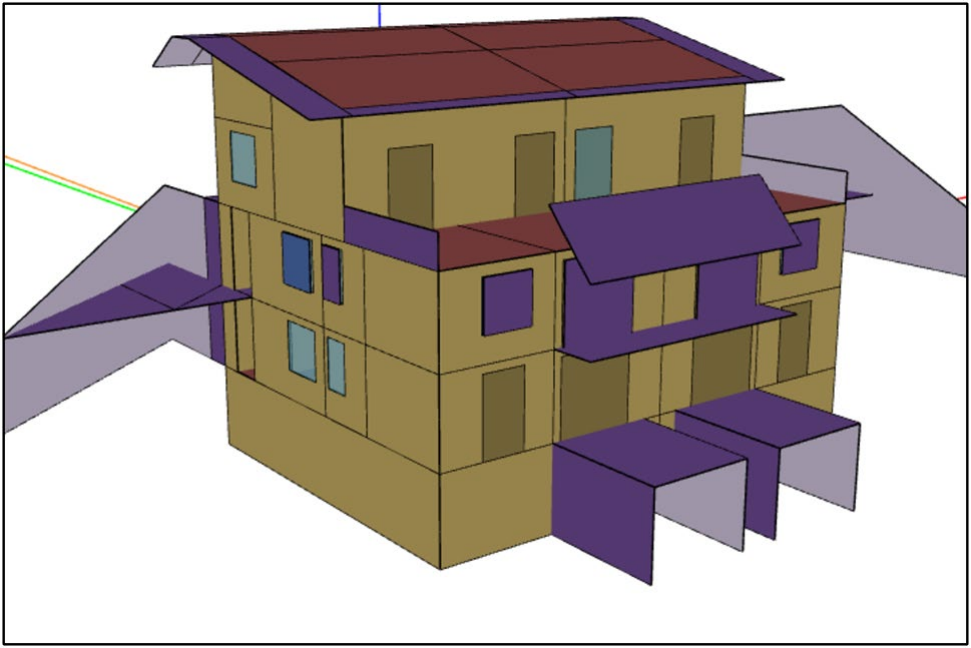
Leaf house building model in openstudio.

The Leaf House is going to serve as the case study building, which is located in Angeli di Rosora, Ancona, Italy. It is a residential apartment building owned and managed by the Loccioni Group (Fig. 2). Serving as a platform for research and innovation, the building places significant emphasis on energy efficiency and sustainable practices. The structure showcases innovative bioclimatic design principles combined with advanced technological systems, and it encompasses six highly insulated residential units within a total area of approximately 470 m^2^.

Several advanced features have been integrated into the Leaf House, including a ventilated roof, solar tubes, intelligent monitoring and control systems, building-integrated photovoltaic (BIPV) modules, geothermal air preconditioning coupled with heat pumps, solar thermal collectors, electrical energy storage systems, and an intuitive energy management interface for occupants.

The building envelope incorporates external walls with a U-value of 0.41 W/m^2^K and windows with U-values between 0.73 and 1.49 W/m^2^K. The HVAC system includes three geothermal heat pumps with integrated air preconditioning and heat recovery connected to a radiant floor heating and cooling system. The heat pumps have heating COP values ranging from 2.9 to 4.6 and cooling EER values between 1.9 and 3.6. Furthermore, seven solar thermal collectors covering a total area of 19 m^2^ are linked to a 1000-L thermal storage tank, supported by a 15 kW electric heater for domestic hot water and heating needs.

Additionally, the building features a rooftop photovoltaic system with a capacity of 20 kWp, consisting of 115 panels covering 150 m^2^. This photovoltaic installation primarily powers the geothermal heat pumps, substantially decreasing the building’s overall electrical energy requirements. The Leaf House achieves an annual normalized primary energy consumption of 54.4 kWh/m^2^, affirming its effectiveness as a near-zero energy building. These results have been validated through dynamic simulations and actual operational data, confirming the successful integration of renewable energy solutions and sophisticated energy management strategies^23^.

### Weather aging acceleration factor

The weather aging acceleration factor (AFtot) quantifies the relationship between laboratory-induced accelerated weathering conditions and real-world exposure. To determine this factor, two primary influences are considered: ultraviolet (UV) radiation exposure and temperature conditions.

To calculate the total acceleration factor, the following parameters are needed^24^: **Ulab**: Total UV energy in the laboratory, **Unat**: Total UV energy under natural environmental conditions, **Tlab**: Laboratory test temperature (in Kelvin), **Tnat**: Natural outdoor temperature (in Kelvin), **E:** Activation energy specific to the material degradation process, **R:** Universal gas constant (fixed value).

The calculation proceeds through these steps:

1. UV acceleration factor (AFuv) is calculated as: ${AF}_{uv}=\frac{{U}_{lab}}{{U}_{nat}}$ (1)
2. Temperature acceleration factor (AFtemp) is determined using the Arrhenius equation: ${AF}_{temp}={e}^{-\frac{E}{R}(\frac{1}{{T}_{lab}}-\frac{1}{{T}_{nat}})}$ (2)
3. Total acceleration factor (AFtot) combines both factors(1) and (2): ${AF}_{tot}={AF}_{uv}*{AF}_{temp}$(3)

For instance, applying the ISO 4892–2 standard and considering a case study located in Ancona, Italy, the calculated acceleration factor relating laboratory simulation hours to natural exposure hours was determined to be approximately 1:20.6.

### EnergyPlus PCM simulation in buildings

EnergyPlus simulates PCM behavior using either “MaterialProperty: PhaseChange” or “MaterialProperty: PhaseChangeHysteresis”. These require advanced conduction algorithms to accurately represent latent heat behavior during phase transitions^25,26^. Typically, EnergyPlus employs the Conduction Transfer Function (CTF) method as its standard algorithm for calculating conduction heat transfer related to building heating and cooling loads. The CTF approach simplifies computations by calculating surface heat flows in a direct, linear way without needing detailed internal temperature or flux information. However, it assumes material properties remain constant and does not provide detailed internal temperature profiles, limiting its effectiveness for dynamically varying conditions typical of PCM applications.

Alternatively, the Conduction Finite Difference (CondFD) algorithm is specifically tailored for more complex scenarios, including those involving PCMs^25,26^. It expands upon the capabilities of the CTF method by accommodating varying thermal conductivity and changing material properties. CondFD dynamically defines computational nodes within each material layer according to the Fourier stability criterion, making it especially effective for shorter simulation time steps. The “MaterialProperty:PhaseChanging” model defines phase-change behavior at a fixed transition temperature, whereas “” additionally accounts for hysteresis, enabling distinct melting and solidification temperatures during the simulation. In a previous work of ours, the “MaterialProperty:PhaseChangingHysteresis” model showed better simulation results for the PCM than the simple “MaterialProperty:PhaseChanging”^19^.

## Results

This section presents the experimental and simulation results assessing the thermal and optical performance of PCM-integrated gypsum and cement boards, both before and after accelerated weather aging. Measurements include solar reflectance, thermal conductivity, specific heat, emissivity, and latent heat. These data are then used in EnergyPlus simulations to quantify changes in energy performance under real-world conditions.

### Measurements

In Supplementary Table 1 the descriptions and dimensions of both cement (CBF) and gypsum (GBF) samples are shown. There are eighteen samples, two for each different type (gypsum and cement) and for each ss-PCM percentage. The sample measurements are taken before and after the accelerated weather aging. All samples were exposed in a Q-SUN XE-1 Xenon accelerated weathering chamber following the ISO 4892–2 standard protocol. For cement boards, a three-step cycle was applied for 1000 h: (1) 1 h 42 min of light exposure, (2) 18 min of combined light and water spray, and (3) a repeat of step 1. Gypsum boards followed a one-step cycle with continuous UV light exposure for 1000 h. The difference in the steps cycles between cement and gypsum samples is due to the nature of the latter. Initial experiments with water spray step in gypsum samples resulted in a dissolved state where no measurements were able to be taken. Because gypsum boards were aged under light-only cycles to avoid dissolution, the observed degradation emphasizes photolytic/thermal mechanisms and may underrepresent moisture-driven degradation for gypsum in service. According to ISO 4892–2 and the reference climate of Ancona, Italy, an acceleration factor of 1:20.6 was calculated, translating 1000 chamber hours into approximately 2.4 years of natural weathering.

#### Solar reflectance measurements

The solar reflectance (SR) values across the ultraviolet (UV, 280–400 nm), visible (VIS, 400–700 nm), and near-infrared (NIR, 700–2500 nm), and total solar spectral ranges were calculated for all samples before and after accelerated weather aging and are presented in Supplementary Table 2. The weather-induced deviations are included as both absolute and percentage changes.

Spectral reflectance was measured using a Cary 5000 UV–Vis-NIR spectrophotometer equipped with an integrating sphere (DRA-2500), covering the 300–2500 nm range. SR was calculated by numerically integrating the measured spectral reflectance data with respect to the standard solar spectral irradiance, following ASTM E903-12. A Spectralon reference standard was used for instrument calibration. The full spectral reflectance curves are provided in Supplementary Fig. 1.

Among the cement board samples, CBF 0% showed a reduction in total SR from 51.0% to 38.0% after aging, while CBF 30% increased from 43.0% to 49.0%. The gypsum-based samples demonstrated SR increases across most compositions, with GBF 0% rising from 69.0% to 72.0% and GBF 30% from 49.0% to 68.0%. These magnitudes of SR decline for unmodified cement boards are comparable to those reported by Paolini et al^20^, who observed 20–30% reductions in cool-wall reflectance after two years of natural exposure, and by Levinson & Akbari et al^27^, who measured 0.15–0.25 absolute SR losses in Portland cement concrete surfaces under natural weathering. The SR gains seen in gypsum boards are consistent with efflorescence-driven brightening reported by Chwast et al^28^.

A detailed breakdown of spectral band SR values (UV, VIS, NIR) and their corresponding changes are available in Supplementary Table 2. The interpretation of these deviations and associated mechanisms is discussed in Discussion section.

#### Thermal conductivity, diffusivity and specific heat measurements

Thermal conductivity (W/mK), thermal diffusivity (mm^2^/s), and specific heat capacity (MJ/m^3^K) were measured using the Hot Disk TSP 1500 with a Kapton 4922 sensor. Supplementary Tables 3,4,5 provide values before and after accelerated aging and their relative deviations.

In cement-based boards aging induced a notable increase in thermal conductivity for the 0% ss-PCM sample (+ 139.7%), with smaller increases at 10% and 20% ss-PCM content (+ 11.1% and + 4.7%, respectively). In contrast, CBF 30% showed a decrease in conductivity (−31.4%). Similar patterns were seen in thermal diffusivity and specific heat, with 0% ss-PCM showing major increases and ss-PCM-rich samples exhibiting smaller or mixed variations. Such increases are consistent with literature reporting moisture-induced conductivity rises of 100–200% in cementitious materials under wetting/drying cycles^29^, while reductions at high PCM loadings mirror the insulating effects observed by Ricklefs et al.^30^ in PCM-cement composites.

In gypsum-based boards, aging generally reduced thermal conductivity and diffusivity across all ss-PCM levels, while specific heat increased consistently, particularly for the 10–30% ss-PCM samples (up to + 82.2% at 10%). These results align with reports that gypsum’s porous microstructure and hygroscopicity lead to conductivity reductions after microcracking or PCM loss^28,31^ and confirm that moisture sorption and recrystallization can raise heat capacity^29^.

#### Emissivity measurements

Emissivity was measured using the Device & Services AE1 Emissometer with a Scaling Digital Voltmeter RD1, using the transient method. Supplementary Figs. 2,3,4,5 show the measurement curves for all samples before and after weather aging. Numerical results are provided in Supplementary Tables 6,7,8.

For CBF samples, aging led to a 30% increase in emissivity for the 0% ss-PCM control. In contrast, ss-PCM-enhanced CBF samples showed slight decreases of 2.2% (10%), 10.2% (20%), and 10.6% (30%) respectively. For GBF samples, the 0% ss-PCM control exhibited a 9.5% decrease. GBF 10% showed a slight increase of 3.5%, while 15%, 20%, and 30% samples showed decreases of 2.2%, 6.8%, and 7.7%, respectively. Despite these changes, all samples maintained high emissivity (~ 0.90–0.93), matching the range reported for cementitious and gypsum surfaces in Barreira et al.^32^.

#### DSC measurements

DSC measurements were performed using a NETZSCH DSC 214 Polyma system on all ss-PCM-enhanced cement and gypsum samples, both before and after accelerated weather aging. Each measurement followed the temperature program described in the Methodology (0–40 °C at 2 K/min, under N₂ purge, triplicate runs), and enthalpies were obtained by integrating the heat flow peaks to ensure reproducibility. PCM-free samples were not measured, as they contain no latent heat storage.

**Fig. 3:**
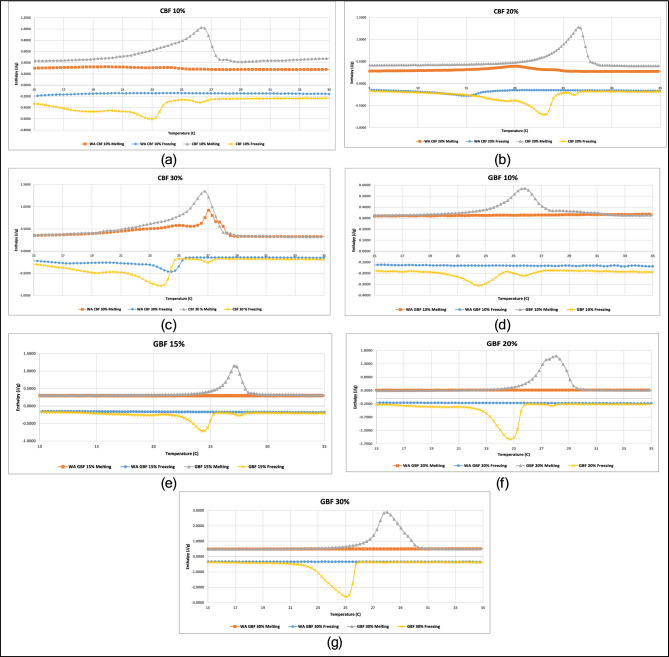
Enthalpy over temperature from dsc measurements, before and after weather aging: (**a**) cbf 10%, (**b**) cbf 20%, (**c**) cbf 30%, (**d**) gbf 10%, (**e**) gbf 15%, (**f**) gbf 20% & (**g**) gbf 30%.

Figure 3 presents the enthalpy over temperature curves for all tested samples before and after aging. A summary of latent heat storage and transition temperatures, used directly as EnergyPlus hysteresis model inputs, is provided in Supplementary Table 9.

For CBF samples, weather aging led to measurable reductions in latent heat and narrower phase change intervals. CBF 10% exhibited a sharp decline in latent heat (~ 79%), with both melting and freezing points shifting to lower temperatures and transition widths narrowing substantially. CBF 20% showed a latent heat reduction of ~ 64%, also accompanied by compressed phase change intervals. CBF 30%, however, retained most of its storage capacity, with a ~ 46% drop in latent heat and less intense change in transition behavior. These levels of enthalpy loss are similar to those reported for microencapsulated PCM composites subjected to 500–700 thermal cycles, where 50–80% degradation was observed^5,33^.

For GBF samples, no detectable latent heat was observed after aging. Prior to exposure, latent heat increased proportionally with ss-PCM content (e.g., ~ 22.52 J/g at 30% ss-PCM), and phase transitions were sharp and consistent. Post-aging measurements revealed complete loss of phase change behavior across all ss-PCM loadings, suggesting extensive PCM degradation or loss within the gypsum matrix. These results highlight a stark contrast between the two matrix types. While cement-based composites retained partial PCM functionality, especially at higher concentrations, gypsum-based boards experienced total degradation of latent heat storage capabilities following environmental exposure, a behavior also reported by Claude et al.^34^ for gypsum plasters under environmental cycling.

The key difference between the two types of boards lies in their composition, as CBF boards contain a large proportion of expanded perlite as aggregate, whereas gypsum boards consist solely of gypsum.

Due to its highly porous structure, expanded perlite can absorb liquid paraffin that leaches out of the PCM matrix during repeated melting and solidification cycles. In contrast, in GBF boards, the continuous phase transitions of PCM, promote its gradual migration toward the specimen surface, ultimately leading to leakage outside the GBF board.

### EnergyPlus simulations

EnergyPlus simulations were conducted for 14 scenarios: a baseline with no intervention (no new material added), one for each ss-PCM-enhanced sample before weather aging, and one after aging. All sample were simulated using the “MaterialProperty:PhaseChangeHysteresis” object, except the aged GBF samples, where PCM functionality was lost and not modeled due to the absence of latent heat, as confirmed by DSC results (Supplementary Table 9). Scenario descriptions are listed in Supplementary Table 10.

The annual total and net energy consumption results are presented in Table 2. Total annual energy consumption refers to simulated end-use loads. Net annual energy equals total site electricity minus onsite PV generation. Prior to accelerated weather aging, all ss-PCM-enhanced materials reduced both total and net annual energy use compared to the baseline. The highest pre-aging energy savings were observed in the GBF 20% scenario, achieving 3.1% total and 5.4% net energy savings. Other notable savings were seen in CBF 30% (2.7% total, 4.8% net) and GBF 30% (2.7% total, 4.8% net). These savings fall within the 4–9% range reported for PCM retrofits in previous simulation studies^35–37^.

**Table 2:** Energyplus simulations results for pcm enhanced samples before and after accelerated weather aging.

Scenarios	Annual energy consumption		Total savings	Net annual energy consumption		Net savings	WA deviation percentage (%)	Samples
	kwh	kwh/m^2^		kWh	kWh/m^2^			
S0	88,677.78	121.62	-	50,894.44	69.80	-	-	-
S1	87,336.11	119.78	1.5%	49,552.78	67.96	2.6%	62.7	CBF 10%
S2	88,177.78	120.94	0.6%	50,394.44	69.12	1.0%		
S3	86,630.56	118.82	2.3%	48,847.22	66.99	4.0%	100.3	CBF 20%
S4	88,683.33	121.63	0.0%	50,900.00	69.81	0.0%		
S5	86,250.00	118.29	2.7%	48,466.67	66.47	4.8%	35.2	CBF 30%
S6	87,105.56	119.47	1.8%	49,322.22	67.65	3.1%		
S7	87,516.67	120.03	1.3%	49,733.33	68.21	2.3%	69.1	GBF 10%
S8	88,319.44	121.13	0.4%	50,536.11	69.31	0.7%		
S9	86,963.89	119.27	1.9%	49,180.56	67.45	3.4%	80.4	GBF 15%
S10	88,341.67	121.16	0.4%	50,558.33	69.34	0.7%		
S11	85,947.22	117.88	3.1%	48,163.89	66.06	5.4%	86.5	GBF 20%
S12	88,308.33	121.12	0.4%	50,525.00	69.30	0.7%		
S13	86,250.00	118.29	2.7%	48,466.67	66.47	4.8%	84.8	GBF 30%
S14	88,308.33	121.12	0.4%	50,525.00	69.30	0.7%		

After accelerated weather aging, energy-saving performance declined across all samples. GBF samples showed the most significant degradation, with post-aging net energy savings dropping to just 0.4% in all cases, representing an ~ 80–97% reduction from their pre-aging values. CBF samples retained more functionality, particularly CBF 30%, which showed a modest drop from 4.8% to 3.1% net savings (a ~ 35% reduction). CBF 30% retained the best post-aging performance (3.1% net savings, a 35% drop from pre-aging), highlighting the relative durability of high-PCM cement boards, as also observed in long-term durability studies of PCM-cement composites^33,34^.

**Fig. 4:**
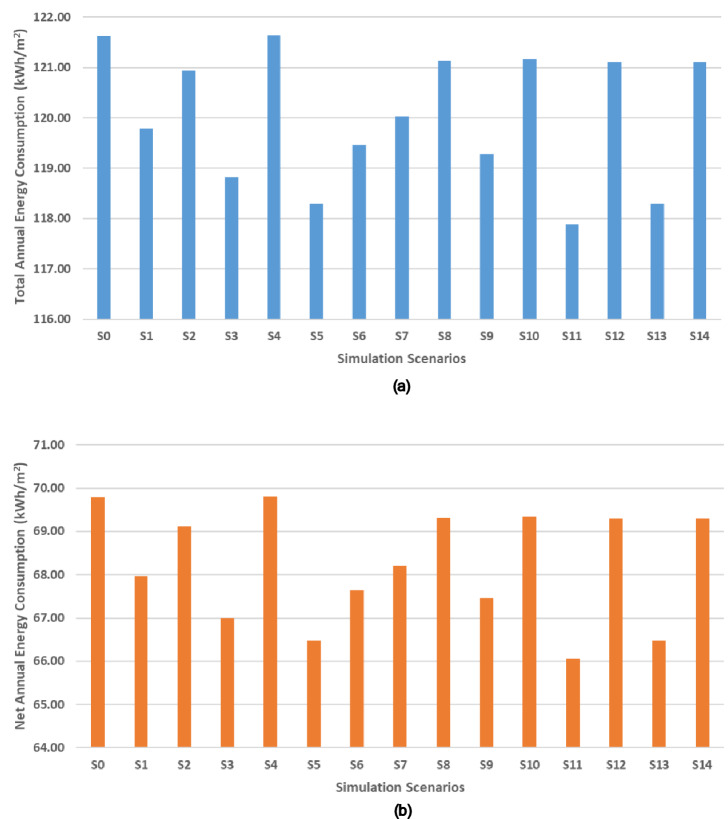
Total (**a**) and Net (**b**) annual energy consumption (kWh/m^2^) EnergyPlus results for all scenarios.

These results highlight that while all ss-PCM-enhanced systems improved energy efficiency prior to aging, only the cement-based boards, particularly those with higher ss-PCM content, maintained measurable performance benefits after environmental exposure. Full simulation results, including savings percentages and weather aging deviation values, are presented in Table 2 and visualized in Fig. 4.

## Discussion

### Accelerated weather aging impact on solar reflectance

**Fig. 5:**
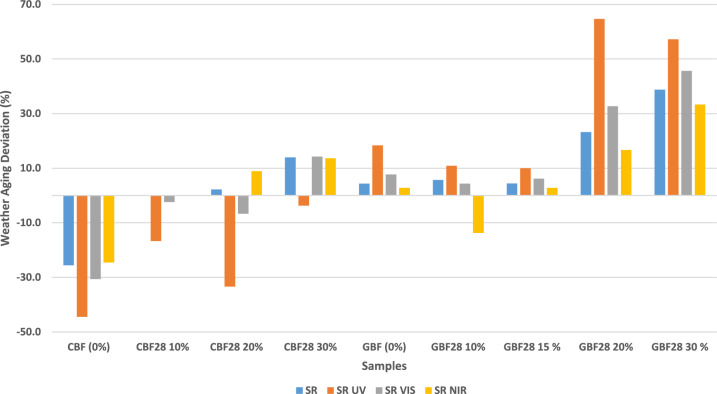
Weather aging deviation of SR, SR-UV, SR-VIS and SR-NIR.

Figure 5 presents the changes in SR due to accelerated weather aging across the UV, VIS, and NIR ranges for both cement and gypsum-based samples with varying ss-PCM content. Distinct trends emerge based on material type and ss-PCM concentration.

The PCM-free cement boards (CBF 0%) exhibited a marked SR reduction of 25.5%, including significant declines in the UV (−44.4%), VIS (−30.6%), and NIR (−24.5%) ranges. These reductions align with typical weathering effects in cementitious materials, such as UV-induced degradation, yellowing, carbonation, and surface soiling^20,27,38^. Paolini et al^20^ reported a 0.20 absolute drop in wall reflectance over four years of natural aging in Milan, causing a 5–11% increase in building cooling demand. Nutakki & Kazim^38^ observed SRI losses from 95–110 to 60–90 after three years of desert exposure, mainly due to dust deposition and photodegradation. Levinson & Akbari^27^ quantified weathering and soiling effects on Portland cement concrete, showing reflectance losses of up to 30% that could be partially reversed by cleaning, confirming that the mechanism involves surface contamination and binder photolysis.

In contrast, ss-PCM-enhanced cement boards showed greater resilience. The CBF 10% sample maintained total SR and NIR values post-aging, with only a 2.4% decrease in VIS and a 16.7% drop in UV reflectance. This UV decline may be linked to the degradation of organic residues or PCM capsule shells that are more sensitive to ultraviolet exposure^16,39,40^. Soudian et al^16^ showed that UV exposure leads to matrix microcracking and binder oxidation in PCM-plasters. Saffari et al^39^ demonstrated that PCM-modified membranes reduce peak thermal stress but still suffer from polymer chain scission under UV. Diebold^40^, found that early chalking of coatings correlates strongly with TiO₂-driven photocatalytic degradation, explaining the formation of loose white residues on weathered PCM surfaces.

Higher ss-PCM contents in cement boards led to overall improvements. CBF 20% showed a modest increase in total SR (+ 2.2%) and NIR (+ 8.9%), while the CBF 30% sample achieved the most notable reflectance gains, with a 14% rise in total SR, 14.3% in VIS, and 13.6% in NIR, and only a minor 3.7% decrease in UV. These enhancements may be attributed to several factors. First, thermal cycling above the PCM melting point (~ 28 °C) could have led to PCM migration or leakage, enhancing diffuse reflectance, particularly in the VIS and NIR regions, due to increased air–solid interfaces and surface roughness^41^. Su et al^41^ demonstrated that PCM layer reconfiguration can increase surface scattering and produce self-switchable radiative cooling effects, consistent with this current work observed gains. Additionally, the breakdown of PCM capsules under UV exposure may have resulted in white particulate residues that increased surface reflectivity, a phenomenon similar to chalking observed in aged coatings^40^. Moreover, the presence of PCM may buffer temperature extremes during weathering, reducing microcracking and surface fouling and thereby preserving optical performance^16,39^. Lastly, the partial loss of paraffin-based PCM, which inherently absorbs in the NIR, may improve the matrix’s native reflectance in that range^42^. Paul et al^42^ reported that paraffin-based PCMs have strong absorptance bands in the 1000–2500 nm region, so their depletion naturally leads to NIR reflectance gains.

These trends suggest that incorporating higher PCM contents in cement boards can mitigate or even reverse reflectance degradation caused by environmental exposure. The CBF 30% sample, with minimal UV loss and significant VIS and NIR gains, exemplifies this behavior and is consistent with earlier observations in studies investigating cool materials enhanced with PCMs^16,39^. Both studies showed that PCM incorporation stabilizes optical properties and can partially compensate for weathering-related losses.

In gypsum-based boards, the unmodified GBF 0% sample showed a moderate increase in reflectance following aging, with a total SR gain of 4.3%, including 18.4% in UV and 7.7% in VIS. This outcome can be attributed to surface recrystallization or efflorescence, where dissolved calcium sulfate re-deposits as a bright, reflective layer during drying^28^. Chwast et al.^28^ documented gypsum efflorescence as a widespread phenomenon that increases surface whiteness and can persist under dry-warm conditions. PCM addition amplified this effect. The GBF 20% and 30% samples demonstrated substantial increases in total SR (+ 23.2% and + 38.8%, respectively), with corresponding VIS improvements of up to 45.7% and NIR gains of 33.3%.

These SR gains may be explained by several mechanisms. Initially, PCM decreases surface reflectance by occupying pores with translucent material. Upon weathering, partial PCM migration or leakage may reopen surface pores and increase air volume, potentially enhancing light scattering, especially in the NIR region^41^. The migration of PCM may also promote localized drying and efflorescence, enhancing the surface’s reflective properties^28^. Additionally, the degradation of capsule shells can leave white residues on the surface, contributing to UV and VIS reflectance enhancement^40,43^. López-Pedrajas et al^43^ showed that nano-encapsulated PCMs can leave fine shell debris after thermal cycling, brightening the composite matrix. Another possible mechanism is surface renewal through PCM phase transitions. The repeated melting and solidification of PCM may induce microstructural changes and surface particle mobilization, potentially mimicking a self-cleaning effect^44^. Lv et al^44^ observed that thermally active foamed concretes can form micro-textures with water-repellent, brightened surfaces over time, resembling a cleaning effect. However, this interpretation remains speculative and requires further experimental validation under real-world exposure conditions.

A notable exception occurred in GBF 10%, where NIR reflectance decreased by 13.7%. This may indicate that at low PCM concentrations, partial redistribution rather than full removal may occur, potentially creating a thin NIR-absorptive layer. This suggests that a threshold PCM concentration may be required to consistently enhance NIR reflectance^42^. These findings highlight material-specific trends in optical response and suggest that ss-PCMs may contribute to reflectance recovery post-aging. While the mechanisms remain speculative, such outcomes could offer additional value in reflective and passive cooling applications^16,43,45^. Xu et al^45^ confirmed that PCM-gypsum boards can reduce surface temperatures and building energy demand, so maintaining or enhancing reflectance post-aging could amplify their passive cooling potential.

### Accelerated weather aging impact on thermal conductivity, diffusivity and specific heat

**Fig. 6:**
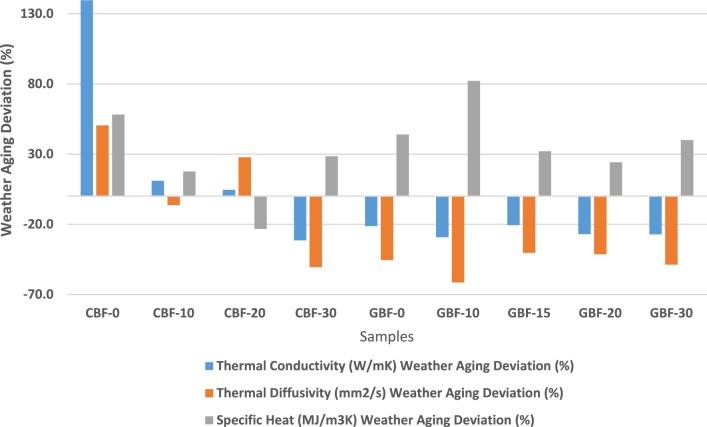
Weather aging deviation (%) of cement and gypsum samples for thermal conductivity, thermal diffusivity and specific heat.

In Fig. 6, the weather aging deviation percentage is depicted over the cement and gypsum samples for thermal conductivity, thermal diffusivity and specific heat. Accelerated weather aging influenced the thermal behavior of ss-PCM-enhanced cement and gypsum boards in distinct ways, depending on material type and PCM concentration. Environmental stressors such as UV radiation, thermal cycling, and moisture exposure affected thermal transport through mechanisms including moisture ingress, PCM redistribution or degradation, and microstructural changes such as densification, cracking, or pore evolution^31,46–48^. Majó et al^46^ reported that repeated thermal cycling leads to a progressive reduction in enthalpy of fatty acid PCMs, indicative of partial phase segregation and leakage, which can alter the effective thermal conductivity of the composite material. Voronin et al^47^demonstrated that higher paraffin loading in microcapsules can thin capsule shells and increase the risk of rupture, potentially changing thermal transport pathways within composites. Taga et al^31^. highlighted gypsum’s photoluminescence sensitivity and microstructural vulnerability under irradiation, which may exacerbate pore evolution during weathering. Erdem et al^48^ showed that mechanical and thermal stresses induce microcracking and densification in cementitious matrices, directly affecting their thermal conductivity and diffusivity.

In cement samples, ss-PCM-free boards exhibited the most significant increases in thermal conductivity and diffusivity after aging. This behavior aligns with known effects of water absorption and continued cement hydration,, both of which enhance thermal transport.

By filling air voids and increasing the solid fraction of the matrix^29^. Szymczak-Graczyk et al^29^ quantified that the thermal conductivity of insulation materials can rise by over 100% under high humidity conditions, demonstrating how moisture ingress dominates thermal behavior. Water’s thermal conductivity, approximately 20 times that of air, plays a dominant role in this increase. In contrast, boards containing ss-PCM showed more stable or even reduced thermal conductivity and diffusivity values, particularly at higher ss-PCM loadings. This indicates that the embedded ss-PCM acted as a thermal buffer, resisting the formation of conductive pathways despite environmental exposure^30^. Ricklefs et al^30^ found that microencapsulated PCM in cement composites reduced thermal conductivity by up to 40% compared to neat cement paste, because PCM microcapsules interrupt heat conduction paths, an effect that persists even under variable moisture content. Specifically, CBF samples with 10–20% ss-PCM showed only moderate changes in thermal properties, suggesting a threshold above which ss-PCM can moderate aging effects without significantly compromising structure. The 30% ss-PCM sample showed a notable decrease in thermal conductivity and diffusivity, that has been associated with ss-PCM redistribution or partial loss during thermal cycling, which reopened pores and increased air volume within the matrix^46,47^. While this could improve insulation performance, it may also indicate microstructural changes or incipient material degradation.

Specific heat values remained generally stable in cement boards across all ss-PCM concentrations, with high-ss-PCM samples maintaining elevated heat capacity even after aging. This confirms previous findings that ss-PCM-cement composites preserve sensible heat storage performance despite surface or microstructural changes^5,30^. Minor specific heat increases in ss-PCM-free CBF boards could be attributed to absorbed moisture or delayed hydration reactions, both of which raise volumetric heat capacity^29^.

In gypsum boards, aging induced a consistent decline in thermal conductivity and diffusivity across all ss-PCM levels, while specific heat increased, particularly at higher ss-PCM contents. This contrasting behavior relative to CBF samples can be attributed to gypsum’s higher porosity and greater sensitivity to surface and structural changes during environmental exposure^28,31^. Chwast et al^28^ noted that gypsum surfaces are highly susceptible to recrystallization and efflorescence under moisture cycling, processes that can occlude or open surface pores and modify thermal transport properties. Reduced conductivity and diffusivity can reflect PCM migration or shell rupture, which left voids or low-density zones in the matrix^46,47^. While potentially beneficial from an insulation perspective, these changes also suggest some degree of PCM loss or phase separation, especially in low- and mid-PCM samples^46^. Nevertheless, the aged gypsum boards maintained or even improved their specific heat values. This trend may result from a combination of moisture absorption, recrystallization phenomena, and the partial retention of PCM^28,29^. Although gypsum matrices are more vulnerable to environmental degradation, especially in the absence of protective coatings, the elevated specific heat after aging suggests that ss-PCM-enhanced boards could still contribute to thermal inertia via sensible storage, even if latent heat functionality is compromised^30^.

Overall, the results demonstrate that ss-PCM-enhanced cement boards maintain thermal conductivity and specific heat more effectively than gypsum boards under accelerated aging. This greater thermal stability supports their suitability for long-term passive regulation in building envelopes.

### Accelerated weather aging impact on emissivity

**Fig. 7:**
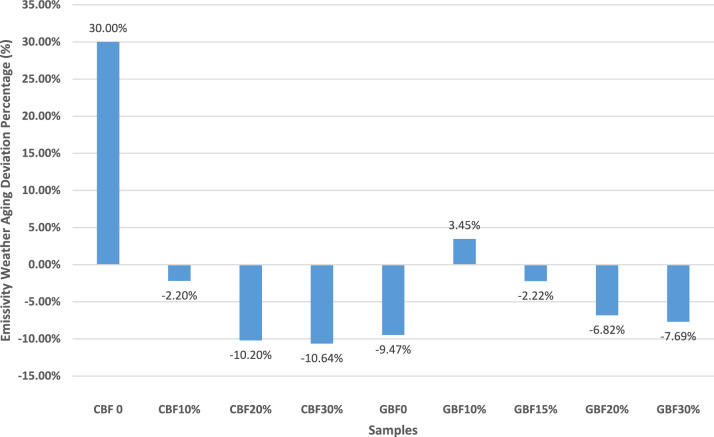
Emissivity weather aging deviation for every sample.

In Fig. 7 the weather aging deviation percentage is depicted over the cement and gypsum samples for the emissivity measurements. Emissivity changes following accelerated weather aging were generally modest across all cement and gypsum samples. Overall, values remained high (~ 0.90), indicating that the thermal radiative performance of both ss-PCM-free and ss-PCM-enhanced materials was largely preserved.

In cement boards, the ss-PCM-free sample exhibited a notable increase in emissivity (~ 30%), that can be associated with moisture uptake, surface roughening, or microstructural densification^32^. Barreira et al.^32^ demonstrated that emissivity is strongly influenced by moisture content, with variations exceeding 10% even for low moisture levels, which supports the interpretation that moisture-related surface changes during weather aging increased emissivity in the control boards. In contrast, ss-PCM-enhanced CBF samples showed small decreases in emissivity with increasing ss-PCM content, ranging from −2.2% to −10.6%. Despite these reductions, the emissivity remained consistently high, suggesting that the ss-PCM addition did not compromise radiative heat emission. Santamouris et al.^49^ reported that cementitious and reflective coatings typically maintain high mid-infrared emissivity (> 0.85) even after prolonged outdoor exposure, which is consistent with the stability observed in this study^32,49^.

For gypsum boards, emissivity responses to aging varied more widely. The ss-PCM-free sample exhibited a moderate reduction (−9.5%), possibly due to surface sealing or partial recrystallization^28,31,32^. Chwast et al.^28^ documented gypsum surface recrystallization during efflorescence, producing smoother, denser surfaces with altered optical properties. Similarly, Taga et al.^31^ found that photoluminescence and structural rearrangements occur in gypsum after UV exposure, potentially modifying surface radiative properties. Barreira et al.^32^ also observed that drying processes can temporarily lower emissivity as surface moisture evaporates, aligning with the reduction seen here. GBF samples with low ss-PCM content (10–15%) showed minor increases or decreases (± 3–7%), while those with 20–30% ss-PCM exhibited reductions of 6.8–7.7%. These trends may be attributed to paraffin migration or surface chalking, especially given gypsum’s higher porosity and sensitivity to moisture^40,43,50^. Diebold^40^ quantified that paint chalking under UV exposure leaves white particulate residues, which could subtly alter emissivity. López Pedrajas et al.^43^ reported that nano-encapsulated PCM gypsum composites exhibit structural changes under repeated phase transitions, including microcracking and PCM shell rupture, which could explain the altered radiative response. Osibodu et al.^50^ reviewed PCM-integrated concretes and found that paraffin migration to the surface can leave transient low-emissivity films that are later removed by oxidation or weathering, which fits the small emissivity changes observed. However, even in these cases, post-aging emissivity remained in the 0.90–0.93 range, aligning with the expected behavior of exposed gypsum surfaces, which are inherently highly emissive^32^.

Importantly, no sample exhibited a decrease indicative of surface contamination by low-emissivity residues (e.g., residual paraffin). This suggests that any ss-PCM that migrated to the surface during aging that can be associated to evaporation, oxidation, or that it was removed by UV and moisture exposure^16,43,50^. Soudian et al.^16^ showed that prolonged UV exposure on PCM-modified plasters leads to surface bleaching and residue removal, eventually restoring high emissivity. López Pedrajas et al.^43^ similarly observed that nano-PCM residues on gypsum surfaces were minimized after repeated wetting and drying cycles. These findings are consistent with previous reports showing that ss-PCM degradation predominantly affects thermal storage properties, while emissivity tends to remain stable or improve slightly due to surface microstructural changes^50^.

Overall, the high and stable emissivity values observed after aging reinforce the radiative durability of ss-PCM-enhanced boards. This behavior is advantageous for passive cooling applications, as it enables continuous thermal radiation to the sky, particularly during night-time conditions. While these results are promising, they are specific to the tested ss-PCM formulations and matrix types. Therefore, broader assessments across different encapsulation methods and material systems are recommended to validate long-term performance.

### Accelerated weather aging impact on DSC results

The DSC analysis demonstrated that accelerated weather aging significantly reduced the latent heat storage capacity of ss-PCM-enhanced materials, with gypsum boards exhibiting the most severe degradation (Fig. 3). In the cement samples, the extent of degradation was closely related to ss-PCM content. For samples with 10–20% ss-PCM, latent heat declined by 64–79%, accompanied by narrower melting/freezing curves and downward shifts in transition temperatures (Supplementary Table 9). These changes suggest partial PCM loss, encapsulation breakdown, or migration within the matrix, all signs of weakened phase change functionality^5,33^. Wadee et al.^5^ showed that even after 700 thermal cycles, paraffin-based PCMs in gypsum plaster and cement mortar exhibited up to 57% latent heat loss, mainly attributed to leakage of PCM from granules rather than chemical decomposition, supporting our observation of enthalpy decline. Jha et al.^33^ reviewed PCM integration strategies and highlighted that high PCM contents and robust encapsulation can delay thermal property degradation by embedding PCM deeper within the matrix, consistent with the improved retention observed in our CBF 30% sample.

By contrast, the CBF 30% sample retained ~ 54% of its original latent heat, with more mild changes in thermal response, indicating that higher PCM content can buffer weather-induced stresses, potentially by embedding more PCM deeper in the matrix or retaining leaked PCM within microstructures^33,34^. Claude et al.^34^ experimentally demonstrated that gypsum plasters with high microencapsulation density preserved a greater fraction of latent heat after repeated thermal cycles, supporting the notion that higher PCM loadings improve resilience.

Conversely, all GBF samples lost measurable latent heat capacity after aging, despite having well-defined phase change behavior before exposure (e.g., up to ~ 22,5 J/g at 30% ss-PCM). The complete absence of a DSC signal post-aging points to PCM evaporation, degradation, or migration out of the gypsum matrix, likely accelerated by gypsum’s high porosity, hygroscopic behavior, and lower structural integrity compared to cement^5,33^. Zhuk^51^ emphasized that durability assessments focused solely on thermal cycling underestimate real-world performance losses, as UV exposure, moisture, and combined weathering accelerate PCM depletion, exactly what is observed here.

These results are consistent with prior findings. For example, Wadee et al.^5^ reported latent heat reductions of 18–57% in cement and gypsum composites after 700 thermal cycles, with thermal conductivity and specific heat remaining largely unchanged, suggesting enthalpy is the most sensitive indicator of long-term PCM performance. Jha et al.^33^ stressed that PCM degradation is often a combination of physical leakage, shell rupture, and chemical oxidation, recommending the use of spectroscopic or FTIR confirmation alongside DSC.533 However, the complete failure of the GBF boards observed here indicates that realistic environmental exposure, combining UV radiation, moisture, and thermal cycling, can lead to more severe degradation than thermal cycling alone, which many durability studies tend to focus on^34,51^.

To address this, recent literature has proposed multiple solutions. For instance, encapsulation with UV- and water-resistant shells, such as melamine–formaldehyde or silica^33^,shape-stabilized PCMs using porous carriers like perlite or diatomite^52^, matrix reinforcement with additives (e.g., carbon fibers) to limit cracking and PCM leakage^53^, and surface coatings to protect from environmental ingress and mechanical degradation. These strategies may be essential for ensuring PCM retention in matrices like gypsum, which otherwise suffer rapid degradation under outdoor exposure.

In summary, cement boards with higher ss-PCM contents demonstrated some resilience after accelerated weather aging, retaining a part of their latent heat capacity and thermal functionality. Gypsum boards, in contrast, exhibited complete loss of phase change behavior following UV exposure, highlighting the matrix’s lower durability even in the absence of moisture stress. This underscores the need to adapt encapsulation strategies based on matrix type and degradation risk. While latent heat was lost in gypsum, the preserved specific heat suggests that limited sensible thermal buffering may still persist.

### Accelerated weather aging impact on building energy simulation

The EnergyPlus simulations showed that applying ss-PCM-enhanced gypsum and cement boards can reduce building energy demand under standard conditions. As shown in Fig. 4, all ss-PCM-enhanced scenarios before accelerated weather aging demonstrated reductions in both total and net annual energy consumption compared to the baseline (S0). The highest energy savings were observed for the unaged GBF 20% sample (S11), with 3.1% total and 5.4% net savings. These results are consistent with previous simulation studies reporting annual energy reductions of 4–9% when PCMs are integrated into wallboards or plasters across various climate zones^17,35–37^. For example, Ahangari & Maerefat^35^ demonstrated 4–7% cooling energy savings with PCM wallboards in four distinct climates, while Aghoei et al.^37^ quantified up to 9% annual energy savings for PCM-integrated opaque envelopes through parametric simulations. Even greater savings are possible with optimized placement strategies, such as combining PCM wallboards with PCM floor systems to maximize diurnal heat storage^17,35–37^^[,54^.

While the aging results indicate severe degradation in PCM functionality, especially in gypsum boards, it is important to interpret these outcomes in the context of the extreme conditions applied (see Methodology). The ISO protocol used in this study reproduces UV, moisture, and thermal cycling stresses more severe than typical field exposure, thereby providing a strict resilience benchmark rather than a direct prediction of service life^20–22^. Paolini et al.^20^ similarly combined natural aging of reflective wall paints with building energy simulation, showing that even modest reflectance losses (≈10–20%) can cause measurable cooling energy penalties over time. More recently, Liu et al.^21^ and Hidalgo-Araujo et al.^22^ evaluated thermochromic and TiO₂-enhanced coatings under accelerated aging, confirming that laboratory protocols can capture long-term optical and thermal performance trends relevant to building energy use.

**Fig. 8:**
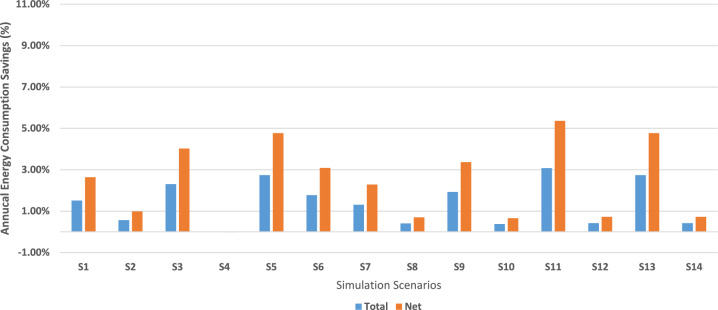
Annual energy consumption total and net savings (%) for all simulation scenarios.

However, the energy-saving performance sharply deteriorated after weather aging. As indicated in Fig. 4, most scenarios with accelerated weather aging (S2, S4, S6, S8, S10, S12, S14) exhibited energy consumption nearly equal to, or exceeding, that of the baseline, underscoring the impact of PCM degradation. Notably, aged CBF 20% (S4) consumed slightly more energy than the baseline, suggesting that when phase change functionality is significantly degraded, PCM inclusion may not just lose effectiveness but even act counterproductively by altering thermal mass and shifting heat release into occupied hours, a phenomenon also reported in PCM design sensitivity analyses^35,55^. Another remark about this sample is that the melting and freezing points shifted, based on the DSC results, such that the phase transition occurred outside the building’s operational temperature range (heating setpoint: 20 °C, cooling setpoint: 26 °C), thereby reducing the material’s ability to buffer temperature fluctuations effectively during periods of peak demand. This trend is quantified in Fig. 8, where the percentage of total and net energy savings before and after aging is presented. The most resilient performance came from the CBF 30% sample (S6), which retained 3.1% net savings, a relatively small drop from its pre-aging value of 4.8%. In contrast, all GBF experienced a severe reduction in energy savings, consistent with the complete loss of latent heat capacity after aging.

**Fig. 9:**
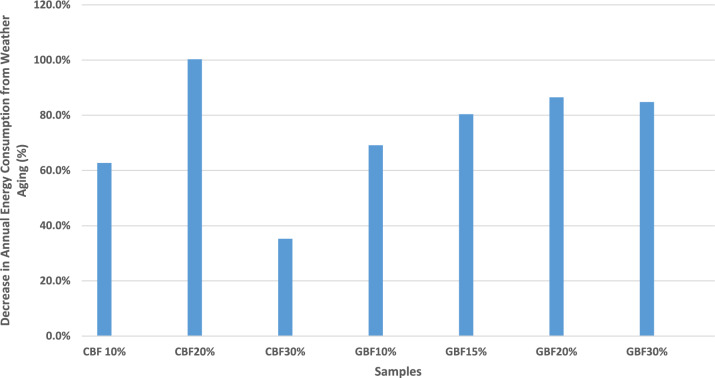
Decrease in total and net annual energy consumption from accelerated weather aging over samples.

Fig 9 further emphasizes this point, showing a loss of ~ 70–87% in energy savings for aged GBF samples. These results correlate with the DSC findings, where no latent heat was detected post-aging in gypsum boards, leading to their simulation without PCM functionality. Cement-based samples, particularly those with higher ss-PCM content, exhibited more moderate declines, with the CBF 30% sample maintaining the highest post-aging performance.

These outcomes highlight the critical role of matrix composition and PCM containment in long-term performance. The cement matrix appears to provide superior protection against environmental degradation, potentially due to stronger matrix-PCM interactions or improved encapsulation integrity^33,34^. These findings echo the conclusions of Wadee et al.^5^, who reported that cement-PCM composites exhibited better retention of thermal capacity after 700 thermal cycles compared with gypsum-PCM composites^5^.

The observed pre-aging savings in this study (1–3% total; 2–5% net) are comparable to ranges reported for passive PCM retrofits in residential buildings^35,55^ The highest pre-aging value (5.4% net in GBF 20%) reflects favorable phase-change temperature matching to the building’s thermal loads, as similarly discussed by Soares et al.^55^. Yet, the sharp post-aging decline highlights a gap in most PCM literature, where durability is often assessed using thermal cycling alone and fails to consider synergistic degradation from UV and moisture exposure^34,51^. This study bridges that gap by combining accelerated weather aging with building simulation to quantify the performance penalty.

In conclusion, while ss-PCM-enhanced envelopes can offer measurable energy savings, their real-world viability depends on long-term environmental durability. Without protective strategies, the materials may lose their phase change effectiveness, diminishing energy-saving benefits. The sustained performance of the CBF 30% sample suggests that material formulation, particularly matrix selection and PCM encapsulation, must be optimized to ensure resilience. Incorporating weather-aging degradation pathways into future simulations is essential to support reliable retrofit planning and material design. While accelerated weather aging enables rapid durability assessment, its intensified UV, moisture, and thermal stressors may trigger degradation pathways that exceed those encountered in real-world conditions^56–60^. This is particularly relevant for the severe PCM loss observed in gypsum boards. Frigione & Rodríguez-Prieto^56^ stress that accelerated aging should always be complemented by field validation. Duan et al.^57^ proposed combined modeling–experimental frameworks to predict service life under real exposure, an approach that could be adopted in future PCM research. To ensure realistic performance predictions, future studies should complement accelerated protocols with long-term field validation and incorporate degradation-informed parameters in building simulations.

### Limitations and future work

While this study provides a comprehensive evaluation of ss-PCM-enhanced gypsum and cement boards under xenon-arc accelerated aging, certain limitations must be acknowledged.

The percentage loading in gypsum and cement boards refers to the incorporated ss-PCMs (shape-stabilized composites), not to pure PCM. For gypsum boards, wt.% was used, while for cement boards vol.% was selected. This distinction arises because the foam carriers used in cement boards occupy a large relative volume, and additions above ~ 23 wt.% would lead to significant deterioration of mechanical properties. To enable comparability and avoid confusion, cement board content was therefore expressed in vol.%. The corresponding ss-PCM mass in the cement boards, expressed in wt.%, is approximately 70% of the reported vol.% addition. Furthermore, no cement board with an intermediate 15% vol. loading was fabricated, leaving a gap in the dataset.

The protocol reproduces solar spectral distribution, temperature, and periodic wetting under controlled conditions, but it does not capture the full complexity of natural outdoor exposure, which also involves fluctuating humidity, pollutant deposition, freeze–thaw cycling, and biological growth. Relative humidity was not actively controlled during testing, and moisture exposure was limited to periodic spray cycles for cement boards. Gypsum boards were aged under light-only conditions to avoid dissolution, which means moisture-driven degradation pathways are underrepresented.

In EnergyPlus, the PCM-enhanced boards were modeled as interior wall layers shielded from direct environmental exposure. Under this configuration, only thermal properties (thermal conductivity, specific heat, latent heat capacity) influence building energy performance. Changes in optical properties such as solar reflectance or emissivity, although measured experimentally, do not affect the simulation outcomes because interior layers are not exposed to solar radiation. Combined with the conservative weathering protocol, this setup emphasizes worst-case durability impacts while omitting optical effects on building energy demand.

Future studies should aim to validate the findings of this work under real outdoor weather conditions, since the present results are based on a standardized accelerated aging protocol (ISO 4892–2). Comparing accelerated aging with long-term natural exposure across different climates would help confirm degradation pathways and refine the correlation between laboratory and field performance. In addition, testing alternative or complementary accelerated weathering standards could further benchmark the durability of PCM-integrated boards. These efforts would provide valuable input for improving predictive models and ensuring that simulation parameters used in tools such as EnergyPlus reflect realistic service conditions.

## Conclusions

This study evaluated the performance and durability of ss-PCM-enhanced gypsum and cement boards subjected to accelerated weather aging and assessed their impact on building energy performance through material characterization and EnergyPlus simulations. Accelerated aging significantly altered thermal properties, reducing PCM functionality and associated energy savings, underscoring the necessity of durability-aware simulations. Notably, PCM layers were simulated as interior components protected from direct weathering, whereas the accelerated aging applied harsher-than-realistic environmental stress. These results therefore reflect a conservative assessment of long-term material durability, emphasizing the need for future field validation.

Cement boards, particularly those with 30% ss-PCM, demonstrated partial resilience, retaining ~ 54% of their latent heat and preserving 3.1% net annual energy savings post-aging. In contrast, gypsum boards lost nearly all latent heat capacity, with energy savings dropping from 5.4% to 0.7%. These findings underscore the critical influence of matrix composition and encapsulation quality on long-term PCM performance and energy efficiency. While standardized accelerated aging provides a valuable stress test for material screening, it cannot fully replicate the complexity of natural degradation over time or across climates.

Overall, ss-PCM-enhanced cement boards show potential promise as durable, passive energy-saving components in warm-climate buildings. Future research should focus on improved encapsulation strategies, real-world exposure validation, and dynamic simulation tools that incorporate degradation pathways. Integrating such durability-aware insights into material development and modeling workflows will be essential to unlock the full potential of ss-PCM-enhanced building envelopes in sustainable construction.

## Supplementary Information

Supplementary Information.

## Data Availability

Processed data and selected raw data supporting the findings of this study are provided in the Supplementary Information. Additional raw datasets are available from the corresponding author upon reasonable request.
