# Supplementary material for: Impact of accelerated weather aging on building energy efficiency using cement and gypsum boards with shape-stabilized phase change materials
Source: Sci Rep. 2025 Nov 19;15:40884. doi: 10.1038/s41598-025-24728-8 (PMC12630816; doi:10.1038/s41598-025-24728-8)
Supplement: Supplementary file 1 — Supplementary Information. [file 41598_2025_24728_MOESM1_ESM.docx]

# Supplementary Information

Supplementary Table 1: Cement (CBF) and Gypsum (GBF) Perlite Samples and their Dimension

| Sample Name | Sample Description | Dimensions (mm*mm*mm) |
| --- | --- | --- |
| CBF (0%) | Cement Board with 0% octadecane | 95*43*40 |
| CBF28 10% | Cement Board with 10% octadecane | 100*45*43 |
| CBF28 20% | Cement Board with 30% octadecane | 100*45*38 |
| CBF28 30% | Cement Board with 30% octadecane | 100*49*38 |
| GBF (0%) | Gypsum Board with 0% octadecane | 100*48*12 |
| GBF28 10% | Gypsum Board with 10% octadecane | 99*49*12 |
| GBF28 15 % | Gypsum Board with 15% octadecane | 99*48*12 |
| GBF28 20% | Gypsum Board with 20% octadecane | 100*48*12 |
| GBF28 30 % | Gypsum Board with 30% octadecane | 95*48*12 |

Supplementary Table 2: Solar Reflectance calculated for Baseline and Weather Aged samples

| **Sample** | **Baseline Samples** | | | | **Weather Aged** | | | | **Weather Aging Deviation** | | | | **Weather Aging Deviation (%)** | | | |
| --- | --- | --- | --- | --- | --- | --- | --- | --- | --- | --- | --- | --- | --- | --- | --- | --- |
|  | SR (%) | SR UV (%) | SR VIS (%) | SR NIR (%) | SR (%) | SR UV (%) | SR VIS (%) | SR NIR (%) | SR (%) | SR UV (%) | SR VIS (%) | SR NIR (%) | SR (%) | SR UV (%) | SR VIS (%) | SR NIR (%) |
| **CBF (0%)** | 51.0 | 36.0 | 49.0 | 53.0 | 38.0 | 20.0 | 34.0 | 40.0 | -13.0 | -16.0 | -15.0 | -13.0 | -25.5 | -44.4 | -30.6 | -24.5 |
| **CBF28 10%** | 45.0 | 30.0 | 42.0 | 48.0 | 45.0 | 25.0 | 41.0 | 48.0 | 0.0 | -5.0 | -1.0 | 0.0 | 0.0 | -16.7 | -2.4 | 0.0 |
| **CBF28 20%** | 45.0 | 33.0 | 45.0 | 45.0 | 46.0 | 22.0 | 42.0 | 49.0 | 1.0 | -11.0 | -3.0 | 4.0 | 2.2 | -33.3 | -6.7 | 8.9 |
| **CBF28 30%** | 43.0 | 27.0 | 42.0 | 44.0 | 49.0 | 26.0 | 48.0 | 50.0 | 6.0 | -1.0 | 6.0 | 6.0 | 14.0 | -3.7 | 14.3 | 13.6 |
| **GBF (0%)** | 69.0 | 49.0 | 65.0 | 72.0 | 72.0 | 58.0 | 70.0 | 74.0 | 3.0 | 9.0 | 5.0 | 2.0 | 4.3 | 18.4 | 7.7 | 2.8 |
| **GBF28 10%** | 70.0 | 55.0 | 69.0 | 73.0 | 74.0 | 61.0 | 72.0 | 63.0 | 4.0 | 6.0 | 3.0 | -10.0 | 5.7 | 10.9 | 4.3 | -13.7 |
| **GBF28 15 %** | 68.0 | 50.0 | 65.0 | 71.0 | 71.0 | 55.0 | 69.0 | 73.0 | 3.0 | 5.0 | 4.0 | 2.0 | 4.4 | 10.0 | 6.2 | 2.8 |
| **GBF28 20%** | 56.0 | 34.0 | 52.0 | 60.0 | 69.0 | 56.0 | 69.0 | 70.0 | 13.0 | 22.0 | 17.0 | 10.0 | 23.2 | 64.7 | 32.7 | 16.7 |
| **GBF28 30 %** | 49.0 | 35.0 | 46.0 | 51.0 | 68.0 | 55.0 | 67.0 | 68.0 | 19.0 | 20.0 | 21.0 | 17.0 | 38.8 | 57.2 | 45.7 | 33.3 |

Supplementary Figure 1: Baseline & Weather Aged (WA) Reflectance (%) at: 200-2500 wavelength (nm) for a) CBF 0%, b) CBF 10%, c) CBF 20%, d) CBF30%, e) GBF 0%, f) GBF 10%, g) GBF 15%, h) GBF 20%, i) GBF 30%

Supplementary Table 3: Hot Disc Measurements for Baseline Samples

| Samples | Thermal Conductivity (W/mK) | | Thermal Diffusivity (mm^2^/s) | | Specific Heat (MJ/m^3^K) | |
| --- | --- | --- | --- | --- | --- | --- |
|  | Average | STD | Average | STD | Average | STD |
| CBF-0 | 0.063 | 0.001 | 1.11 | 0.09 | 0.0574 | 0.0050 |
| CBF-10 | 0.063 | 0.001 | 1.11 | 0.09 | 0.0574 | 0.0050 |
| CBF-20 | 0.086 | 0.002 | 0.90 | 0.24 | 0.1017 | 0.0223 |
| CBF-30 | 0.086 | 0.007 | 1.15 | 0.35 | 0.0811 | 0.0261 |
| GBF-0 | 0.170 | 0.001 | 1.72 | 0.11 | 0.0997 | 0.0063 |
| GBF-10 | 0.164 | 0.001 | 1.84 | 0.17 | 0.0894 | 0.0069 |
| GBF-15 | 0.175 | 0.002 | 1.81 | 0.13 | 0.0972 | 0.0066 |
| GBF-20 | 0.170 | 0.003 | 1.72 | 0.10 | 0.0988 | 0.0049 |
| GBF-30 | 0.173 | 0.001 | 1.56 | 0.12 | 0.1120 | 0.0085 |

Supplementary Table 4: Hot Disc Measurements for Weather Aged Samples

| Samples | Thermal Conductivity (W/mK) | | Thermal Diffusivity (mm^2^/s) | | Specific Heat (MJ/m^3^K) | |
| --- | --- | --- | --- | --- | --- | --- |
|  | Average | STD | Average | STD | Average | STD |
| CBF-0_WA | 0.151 | 0.002 | 1.67 | 0.08 | 0.0908 | 0.0036 |
| CBF-10_WA | 0.070 | 0.0005 | 1.04 | 0.01 | 0.0675 | 0.0004 |
| CBF-20_WA | 0.090 | 0.001 | 1.15 | 0.01 | 0.078 | 0.0004 |
| CBF-30_WA | 0.059 | 0.001 | 0.57 | 0.03 | 0.1042 | 0.0030 |
| GBF-0_WA | 0.134 | 0.001 | 0.94 | 0.02 | 0.1436 | 0.0019 |
| GBF-10_WA | 0.116 | 0.002 | 0.71 | 0.02 | 0.1629 | 0.0020 |
| GBF-15_WA | 0.139 | 0.002 | 1.08 | 0.02 | 0.1284 | 0.0015 |
| GBF-20_WA | 0.124 | 0.001 | 1.01 | 0.02 | 0.1228 | 0.0017 |
| GBF-30_WA | 0.126 | 0.001 | 0.80 | 0.01 | 0.1569 | 0.0013 |

Supplementary Table 5: Weather Aging Deviation

| Samples | Thermal Conductivity (W/mK) | Thermal Diffusivity (mm^2^/s) | Specific Heat (MJ/m^3^K) | |
| --- | --- | --- | --- | --- |
| Weather Aging Deviation | | | | |
| CBF-0 | +0.088 | +0.56 | | +0.0334 |
| CBF-10 | +0.007 | -0.07 | | +0.0101 |
| CBF-20 | +0.004 | +0.25 | | -0.0237 |
| CBF-30 | -0.027 | -0.58 | | +0.0231 |
| GBF-0 | -0.036 | -0.78 | | +0.0439 |
| GBF-10 | -0.048 | -1.13 | | +0.0735 |
| GBF-15 | -0.036 | -0.73 | | +0.0312 |
| GBF-20 | -0.046 | -0.71 | | +0.024 |
| GBF-30 | -0.047 | -0.76 | | +0.0449 |


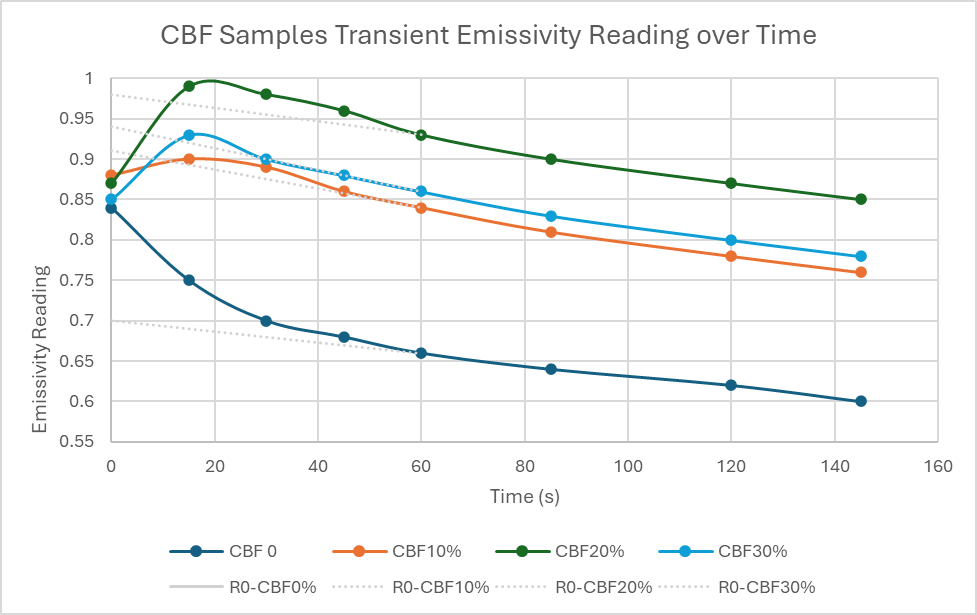


Supplementary Figure 2: Transient Emissivity Reading over time for CBF samples before weather aging


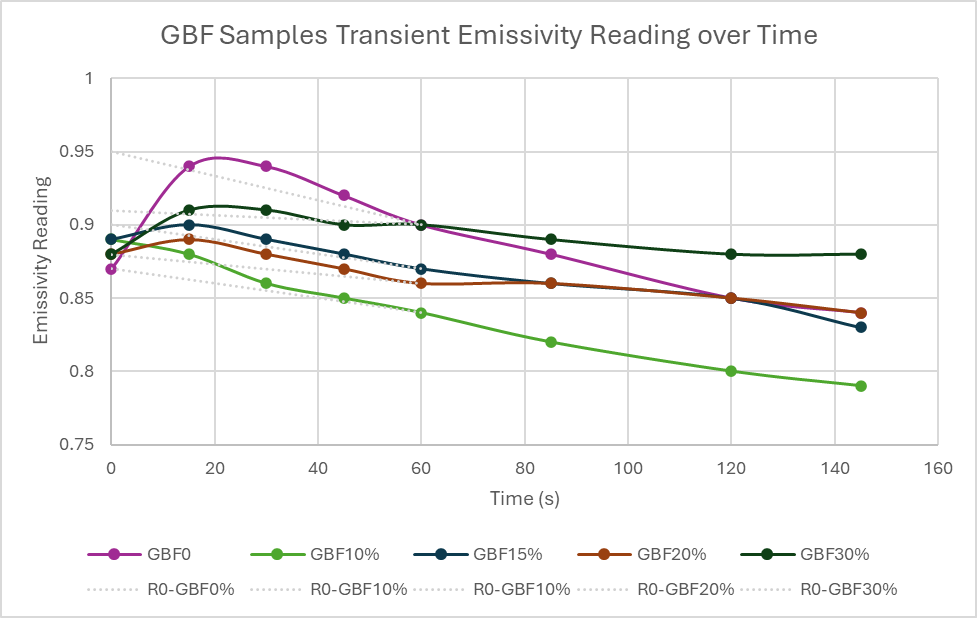


Supplementary Figure 3: Transient Emissivity Reading over time for GBF samples before weather aging

Supplementary Table 6: Emissivity Measurements for CBF and GBF samples before weather aging

| N/N | Time (s) | CBF 0 | CBF10% | CBF20% | CBF30% | GBF0 | GBF10% | GBF15% | GBF20% | GBF30% |
| --- | --- | --- | --- | --- | --- | --- | --- | --- | --- | --- |
| 1 | 0 | 0.84 | 0.88 | 0.87 | 0.85 | 0.87 | 0.89 | 0.89 | 0.88 | 0.88 |
| 2 | 15 | 0.75 | 0.9 | 0.99 | 0.93 | 0.94 | 0.88 | 0.9 | 0.89 | 0.91 |
| 3 | 30 | 0.7 | 0.89 | 0.98 | 0.9 | 0.94 | 0.86 | 0.89 | 0.88 | 0.91 |
| 4 | 45 | 0.68 | 0.86 | 0.96 | 0.88 | 0.92 | 0.85 | 0.88 | 0.87 | 0.9 |
| 5 | 60 | 0.66 | 0.84 | 0.93 | 0.86 | 0.9 | 0.84 | 0.87 | 0.86 | 0.9 |
| 6 | 85 | 0.64 | 0.81 | 0.9 | 0.83 | 0.88 | 0.82 | 0.86 | 0.86 | 0.89 |
| 7 | 120 | 0.62 | 0.78 | 0.87 | 0.8 | 0.85 | 0.8 | 0.85 | 0.85 | 0.88 |
| 8 | 145 | 0.6 | 0.76 | 0.85 | 0.78 | 0.84 | 0.79 | 0.83 | 0.84 | 0.88 |
| E=1*R0 extrapolated (vis) | | **0.7** | **0.91** | **0.98** | **0.94** | **0.95** | **0.87** | **0.9** | **0.88** | **0.91** |
|  | |  |  |  |  |  |  |  |  |  |

**
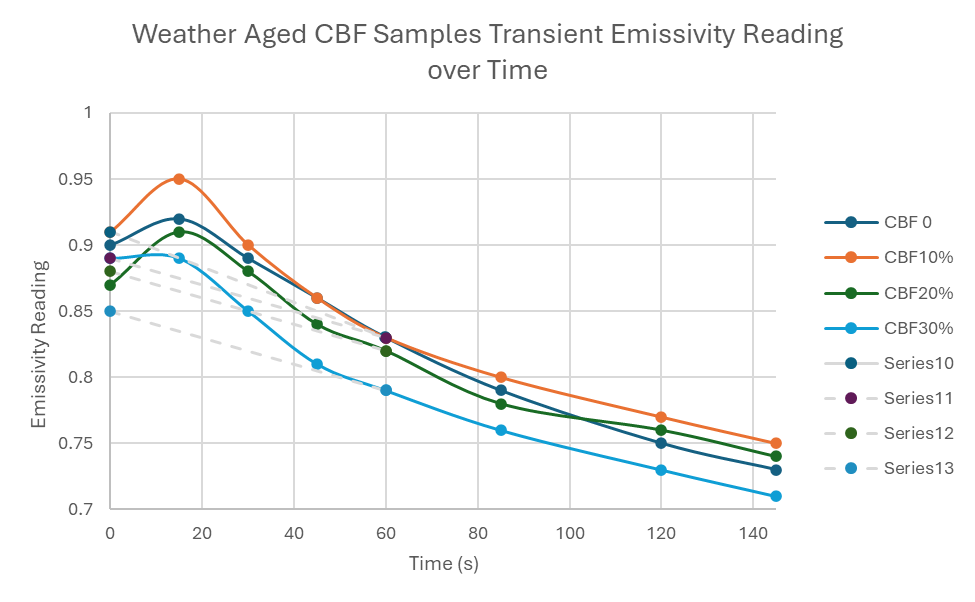
**

Supplementary Figure 4: Transient Emissivity Reading over time for CBF samples after weather aging


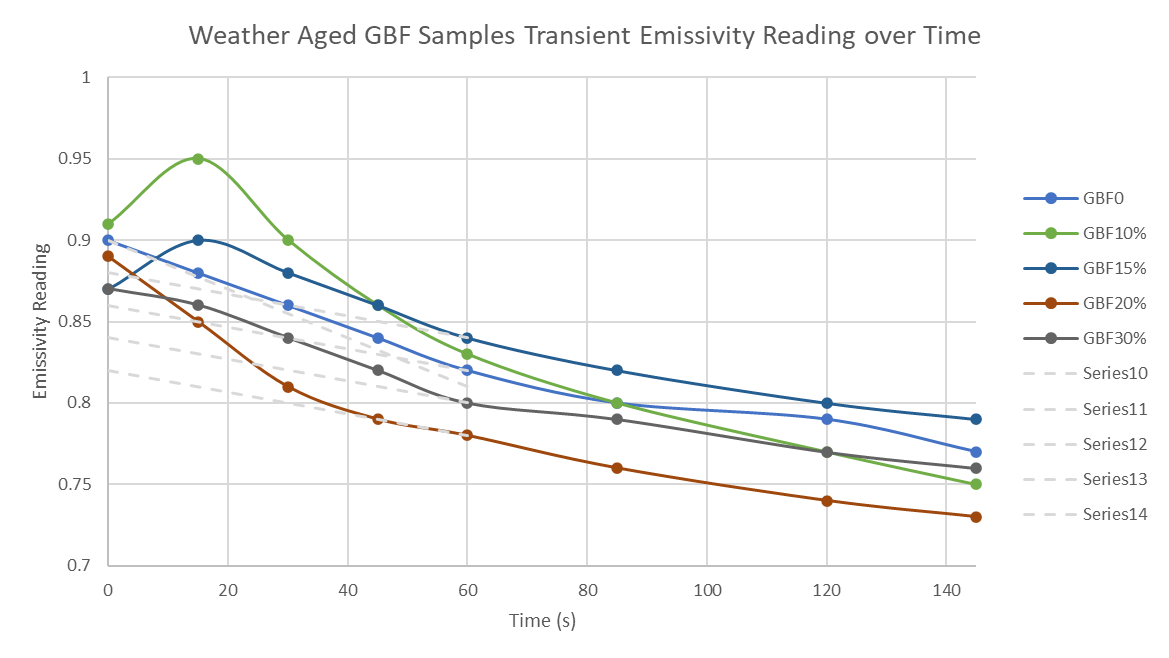


Supplementary Figure 5: Transient Emissivity Reading over time for GBF samples after weather aging

Supplementary Table 7: Emissivity Measurements for CBF and GBF samples after weather aging

| N/N | Time (s) | CBF 0 WA | CBF10% WA | CBF20% WA | CBF30% WA | GBF0 WA | GBF10% WA | GBF15% WA | GBF20% WA | GBF30% WA |
| --- | --- | --- | --- | --- | --- | --- | --- | --- | --- | --- |
| 1 | 0 | 0.9 | 0.91 | 0.87 | 0.89 | 0.9 | 0.93 | 0.87 | 0.89 | 0.87 |
| 2 | 15 | 0.92 | 0.95 | 0.91 | 0.89 | 0.88 | 0.87 | 0.9 | 0.85 | 0.86 |
| 3 | 30 | 0.89 | 0.9 | 0.88 | 0.85 | 0.86 | 0.84 | 0.88 | 0.81 | 0.84 |
| 4 | 45 | 0.86 | 0.86 | 0.84 | 0.81 | 0.84 | 0.82 | 0.86 | 0.79 | 0.82 |
| 5 | 60 | 0.83 | 0.83 | 0.82 | 0.79 | 0.82 | 0.81 | 0.84 | 0.78 | 0.8 |
| 6 | 85 | 0.79 | 0.8 | 0.78 | 0.76 | 0.8 | 0.79 | 0.82 | 0.76 | 0.79 |
| 7 | 120 | 0.75 | 0.77 | 0.76 | 0.73 | 0.79 | 0.77 | 0.8 | 0.74 | 0.77 |
| 8 | 145 | 0.73 | 0.75 | 0.74 | 0.71 | 0.77 | 0.75 | 0.79 | 0.73 | 0.76 |
| E=1*R0 extrapolated (vis) | | **0.7** | **0.91** | **0.89** | **0.88** | **0.85** | **0.86** | **0.9** | **0.88** | **0.82** |

Supplementary Table 8: Emissivity Measurements Weather Aging Deviation for CBF and GBF samples

| Sample | Emissivity Weather Aging Deviation | Emissivity Weather Aging Deviation (%) |
| --- | --- | --- |
| CBF 0 | 0.21 | 30.00% |
| CBF10% | -0.02 | -2.20% |
| CBF20% | -0.1 | -10.20% |
| CBF30% | -0.1 | -10.64% |
| GBF0 | -0.09 | -9.47% |
| GBF10% | 0.03 | 3.45% |
| GBF15% | -0.02 | -2.22% |
| GBF20% | -0.06 | -6.82% |
| GBF30% | -0.07 | -7.69% |

Supplementary Table 9: EnergyPlus MaterialProperty:PhaseChangeHysteresis Inputs for the samples that have PCM content

| **Name** | **Units** | **CBF 10%** | ***WA CBF 10%*** | ***CBF 20%*** | ***WA CBF 20%*** | ***CBF 30%*** | ***WA CBF 30%*** | ***GBF 10%*** | ***GBF 15%*** | ***GBF 20%*** | ***GBF 30%*** |
| --- | --- | --- | --- | --- | --- | --- | --- | --- | --- | --- | --- |
| Latent Heat during the Entire Phase Change Process | J/kg | *6,034* | *1,244* | *11,970* | *4,286* | *14,750* | *8,028* | *5,393* | *6,324* | *13,130* | *22,520* |
| Liquid State Thermal Conductivity | W/mK | *0.063* | *0.07* | *0.086* | *0.09* | *0.086* | *0.059* | *0.164* | *0.175* | *0.17* | *0.173* |
| Liquid State Density | Kg/m^3^ | *510* | *537* | *630* | *627* | *650* | *642* | *870* | *960* | *940* | *950* |
| Liquid State Specific Heat | J/kgK | *106* | *125* | *160* | *124* | *125* | *162* | *103* | *101* | *105* | *118* |
| High Temperature Difference in Melting Curve | deltaC | *2.5* | *2.25* | *2* | *4* | *2.5* | *2* | *5.75* | *2.75* | *2.75* | *3* |
| Peak Melting Temperature | C | *26.31* | *19.78* | *26.53* | *20.03* | *26.80* | *27.03* | *25.82* | *27.06* | *28.09* | *28.04* |
| Low Temperature Difference in Melting Curve | deltaC | *7.5* | *1.75* | *5.5* | *2* | *7.75* | *9.5* | *6.75* | *4* | *5* | *4.75* |
| Solid State Thermal Conductivity | W/mK | *0.063* | *0.07* | *0.086* | *0.09* | *0.086* | *0.059* | *0.164* | *0.175* | *0.17* | *0.173* |
| Solid State Density | Kg/m^3^ | *540* | *537* | *630* | *627* | *650* | *642* | *870* | *960* | *940* | *950* |
| Solid State Specific Heat | J/lgK | *106* | *125* | *160* | *124* | *125* | *162* | *103* | *101* | *105* | *118* |
| High Temperature Difference in Freezing Curve | deltaC | *2* | *3* | *4* | *3* | *1.24* | *1* | *5* | *1.25* | *1.25* | *1.25* |
| Peak Freezing Temperature | C | *23.00* | *17.24* | *22.98* | *15.24* | *23.74* | *24.49* | *22.48* | *24.24* | *24.76* | *25.02* |
| Low Temperature Difference in Freezing Curve | deltaC | *7.5* | *3* | *7.75* | *2.75* | *10.76* | *9.5* | *4.75* | *3.25* | *3.75* | *4* |

Supplementary Table 10: Simulation Scenarios and their Descriptions

| Scenarios | Description |
| --- | --- |
| S0 | Baseline |
| S1 | CBF 10% on external walls and roof |
| S2 | WA CBF 10% on external walls and roof |
| S3 | CBF 20% on external walls and roof |
| S4 | WA CBF 20% on external walls and roof |
| S5 | CBF 30% on external walls and roof |
| S6 | WA CBF 30% in external walls and roof |
| S7 | GBF 10% on external walls and roof |
| S8 | WA GBF 10% on external walls and roof |
| S9 | GBF 15% on external walls and roof |
| S10 | WA GBF 15% in external walls and roof |
| S11 | GBF 20% on external walls and roof |
| S12 | WA GBF 20% on external walls and roof |
| S13 | GBF 30% on external walls and roof |
| S14 | WA GBF 30% on external walls and roof |
